# Effects of continuous renal replacement therapy on serum cytokines, neutrophil gelatinase-associated lipocalin, and prognosis in patients with severe acute kidney injury after cardiac surgery

**DOI:** 10.18632/oncotarget.13254

**Published:** 2016-11-09

**Authors:** Heng-jin Wang, Pei Wang, Nan Li, Cheng Wan, Chun-ming Jiang, Jing-song He, Dong-jin Wang, Miao Zhang, Ling-yun Sun

**Affiliations:** ^1^ Department of Nephrology, Affiliated Drum Tower Hospital, Medical School of Nanjing University, Nanjing, China; ^2^ Nanjing Drum Tower Hospital, Nanjing Medical University (Nanjing University of Chinese Medicine), Nanjing, China; ^3^ Nanjing Maternity and Child Health Hospital, Nanjing Medical University, Nanjing, China; ^4^ Department of Cardiac Surgery, Affiliated Drum Tower Hospital, Medical School of Nanjing University, Nanjing, China

**Keywords:** continuous renal replacement therapy, acute kidney injury, multiple organ dysfunction syndrome, cardiac surgery, cytokines

## Abstract

The aim of our study was to evaluate the effect of continuous renal replacement therapy (CRRT) on serum cytokines, neutrophil gelatinase-associated lipocalin (NGAL), and prognosis in patients with severe acute kidney injury (AKI) following cardiac surgery. A total number of 153 patients with severe AKI following cardiac surgery were treated with CRRT. They were divided into the survival and non-survival groups. Clinical data from these two groups before and after CRRT were recorded and analyzed. It was found that the number of impaired organs, MODS and APACHE II scores were significantly higher in the non-survival group than those in the survival group before CRRT. After CRRT, MODS and APACHE II scores decreased significantly. The post-CRRT levels of serum TNF-a and IL-6 were significantly decreased. After CRRT, serum NGAL decreased in the two groups, but the levels were higher in the non-survival group than those in the survival group. MODS and APACHE II scores could be used to evaluate the severity of AKI in patients after cardiac surgery. CRRT is an effective treatment for these patients and high levels of TNF-a, IL-6, and NGAL are associated with a poor prognosis in these patients.

## INTRODUCTION

Acute kidney injury (AKI) represents a major complication of cardiac surgery [1]. There are up to 50 % of patients undergoing cardiac surgery develop AKI and, despite the improvements in post-operative management of critically ill patients, cardiac surgery-associated AKI (CS-AKI) still carries an increased risk of death [2, 3]. AKI is the strongest risk factor for postoperative mortality, having an odds ratio of 7.9 and a mortality in excess of 60% [4]. Therefore, improving the outcomes for these patients is an urgent necessity. Continuous renal replacement therapy (CRRT), used as a “life support technology,” is very effective and carries a number of advantages in postoperative management of renal insufficiency [5].

The scope of CRRT has expanded from a simple extension of renal replacement therapy to the treatment for multiple organ system support, and it is playing an especially unique role in the treatment of critically ill patients with multiple organ dysfunction syndrome (MODS). CRRT is an effective means of fluid and solute management in AKI [6]. The advantages of treating patients with severe AKI after cardiac surgery with CRRT are as follows: 1) maintaining hemodynamic stability, 2) removal of inflammatory mediators, 3) improvement of immune function, 4) improvement of organ function, 5) providing fluid balance and nutritional support, 6) allowing the reduction of the dosage of vasoactive drugs, and 7) promoting recovery of renal function [7, 8].

Biomarker for CRRT is a largely un-explored area. Neutrophil gelatinase-associated lipocalin (NGAL) has been emerging as a promising biomarker indicating nephrotoxic insults [9]. Cytokines and other inflammatory mediators can influence pathogenesis of post-surgical complications such as septic shock and associated AKI and could become biomarkers for CRRT [5]. Monitoring the levels of these components may be used in early diagnosis, guiding CRRT, measuring progress, and predicting prognosis in patients [9]. There are few reports documenting the effects of CRRT on serum NGAL and cytokines in patients with severe AKI after cardiac surgery.

The objective of the current study is to elucidate the relationship between CRRT and the levels of NGAL and serum cytokines, and the overall impact on patient prognosis.

## RESULTS

### CRRT treatment and outcome

A total of 153 patients with severe AKI after cardiac surgery were treated with CRRT in the blood purification center in our hospital. There were 89 patients in the survival group and 64 patients in the non-survival group. The mortality rate was 41.8%. In the survival group, the renal function of 61 patients recovered after 19-33 days of treatment with CRRT. Hemodialysis for 2-4 weeks was required by 17 patients after stopping the treatment of CRRT. Maintenance dialysis was required by 11 patients. In the non-survival group, 7 patients died from heart failure, 12 died from gastrointestinal bleeding, 8 died from infection, 34 died from multiple organ failure, and 3 died from disseminated intravascular coagulation.

### Effect of CRRT on hemodynamics, respiratory function, and severity of illness

To evaluate the effect of CRRT on these patients, acute physiology and chronic health evaluation II (APACHE II)and MODS scores were monitored. APACHE score is a physiological based classification system used to evaluate acute and chronic health status (Table 1) [10]. MODS was defined as a cardiovascular system dysfunction (systolic blood pressure < 90 mmHg or mean arterial pressure < 70 mmHg), kidney dysfunction (urine output < 0.5 ml/kg body weight/hour for one hour, despite adequate fluid resuscitation), respiratory system dysfunction (ratio of PaO_2_ to FiO_2_ < 250 in the presence of other dysfunctional organs or systems), hematologic dysfunction (platelet count < 80.000/mm^3^ or decreased by 50% in the three days preceding enrollment in the absence of liver cirrhosis or previously known hematological disease), or metabolic dysfunction (unexplained metabolic acidosis: pH < 7.30 or base deficit > 5.0 mmol/L in association with a plasma lactate level > 1.5 times of the upper normal limit) [11]. The APACHE II and MODS scores of the non-survival group were significantly higher than those of the survival group before CRRT. The mean arterial pressure (MAP) and oxygenation index (OI) were lower than in the survival group (P < 0.05). After 24 hours of CRRT treatment, the MAP and OI in both groups improved greatly compared with before treatment. The MODS score, APACHE II score, and the heart rate (HR) of the survival group all decreased significantly (t > 2.045, P < 0.05) (Table 2). These results indicated that CRRT could improve the hemodynamics and respiratory function, and relieve the severity of illness.

**Table 1:** APACHEII scores

age		≤44□0; 45-54□2; 55-64□3; 65-74□≥5						A score			
	+4		+3	+2	+1	0	+1	+2	+3	+4	
temperature (°C)	≥41		39-40.9		38.5-38.9	36-38.4	34-35.9	32-33.9	30-31.9	≤29.9	
Mean rterialpressure (mmHg)	≥160		130-159	110-129		70-109		50-69		≤49	
.heart rate	≥180		140-179	110-139		70-109		55-69	40-54	≤39	
Reapiratory rate	≥50		35-49		25-34	12-24	10-11	6-9		≤5	
PaO2 (mmHg) (FiO2<50%) A-aDO2(FiO2>50%)	≥500		350-499	200-349		>70 <200	61-70 ………	………	55-60 ………	<55 ……	
arterial PH	≥7.7		7.6-7.69		7.5-7.59	7.33-7.49		7.25-7.32	7.15-7.24	<7.15	
SERUM SODIUM (mmol/L)	≥180		160-179	155-159	150-154	130-149		120-129	111-119	≤110	
Serum Potassrum (mmol/L)	≥7		6-6.9		5.5-5.9	3.5-5.4	3-3.4	2.5-2.9		<2.5	
Serum Creatinine (mg/dL)	≥3.5		2-3.4	1.5-1.9		0.6-1.4		<0.6			
Hemtocrit(%)	≥60			50-59.9	46-49.9	30-45.9		20-29.9		<20	
WBC(*1000)	≥40			20-39.9	15-19.9	3-14.9		1-2.9		<1	
Glasgow Coma Scope (GCS)											
Total acutephysiology score(APS)											

**Table 2:** Changes in HR, MAP, and OI in the survival (A) and non-survival (B) groups before and after CRRT (mean ± standard deviation)

	MODS score		APACHE II score		HR (/min)		MAP (mm Hg)		OI	
Groups	A	B	A	B	A	B	A	B	A	B
Before CRRT (pre-CRRT)	6.7 ± 2.3^△^	13.1 ± 3.7	16.9 ± 4.8^△^	26.7 ± 9.9	98.2 ± 16.7	106.1 ± 23.0	73.9 ± 12.1^△^	64.8 ± 11.3	221.4 ± 47.9^△^	169.7 ± 53.9
CRRT 24h	3.9 ± 1.7*	12.8 ± 4.1	9.8 ± 3.1*	25.0 ± 10.8	86.9 ± 19.8*	97.3 ± 16.9	78.1 ± 6.8*	66.9 ± 10.1*	279.3 ± 78.6*	203.1 ± 70.2*

CRRT, continuous renal replacement therapy; MODS, multiple organ dysfunction syndrome; APACHE, acute physiology and chronic health evaluation; HR, heart rate; MAP, mean arterial pressure; OI, oxygenic index; CBP, continuous blood purification

Group A (survival) had 89 cases and Group B (non-survival) had 64 cases. In the same group, CBP at 24 hours compared with pre-CBP, t > 2.045,*P < 0.05; between the two groups, t >1.984, ^∆^ P < 0.05

### Effect of CRRT on renal function, WBC, and PLT

**Figure 1:**
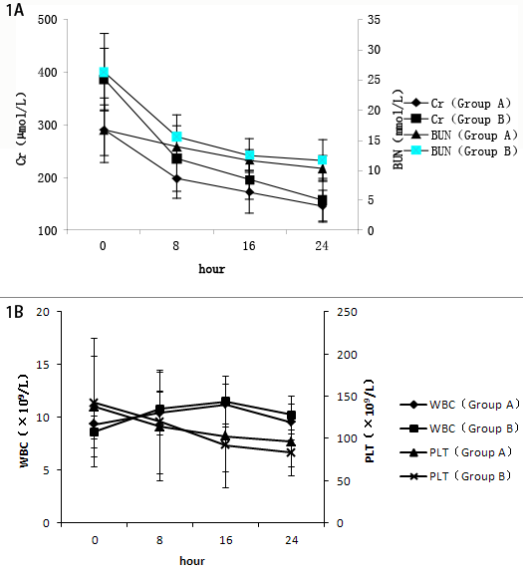
**A.** Changes of renal function in the survival (A) and non-survival (B) groups. In the same group, comparing with 0 h, *P < 0.05, **P < 0.01. **B.** Changes in white blood cells (WBC) count and platelets (PLT) in the survival (A) and non-survival (B) groups. Comparison between groups, *P* > 0.05.

The renal function, WBC and PLT were analyzed before and after CRRT. The serum BUN and Cr of the non-survival group was significantly higher than those of the survival group before treatment (t > 1.984, P < 0.05), indicating that patients in the non-survival group had very poor renal function that were not benefited from CRRT. After the treatment with continuous blood purification for 12, 24, and 48 h, the levels of BUN and Cr in these two groups were significantly lower than the levels before treatment (t > 2.626, P < 0.01). The WBC and PLT count in these two groups were not significantly different before and after treatment (Figure 1A and 1B). These results suggested that CRRT could greatly improve patients’ renal function, although it did not affect the number of blood cells such as WBC or PLT.

### CRRT affects secretion of cytokines

**Figure 2:**
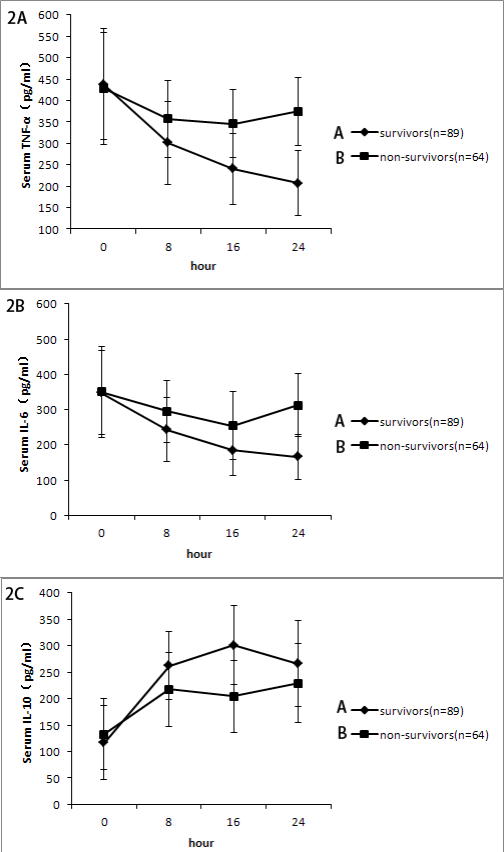
A. Change of serum tumor necrosis factor-alpha(TNF-α) level at different time points Serum TNF-α levels in the survival group was lower than those in the non-survival group at 16h and 24 h(* *P* < 0.05). **B.** Change of interleukin-6 (IL-6) level at different time points(* *P* < 0.05, survival group vs. non-survival group). **C.** Change of interleukin-10(IL-10) level at different time points(*P* > 0.05, survival group vs. non-survival group).

Because cardiac surgery is usually accompanied with inflammatory reactions, the changes of inflammatory mediators were evaluated. It was found that serum pro-inflammatory cytokine TNF-α of the survival group were significantly decreased after CRRT treatment (207.3 ± 75.9 pg/mL vs. 437.6 ± 129.8 pg/mL, P < 0.05). The same pattern was seen with IL-6 (165.9 ± 64.7 pg/mL vs. 350.4 ± 129.3 pg/mL, P < 0.05). These values were not significantly reduced in the non-survival group. There was no significant change of the anti-inflammatory factor, IL-10 in these two groups before and after treatment (Figure 2A-2C).

### Effect of CRRT on NAGL in severe AKI

**Figure 3:**
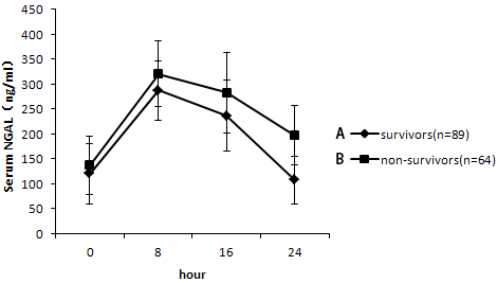
Change of neutrophil gelatinase-associated lipocalin (NGAL) level at different time points Serum NGAL level was higher in the non-survival group (197.4 ± 60.1 ng/mL) than those in the survival group (107.1 ± 48.2 ng/mL) (*P = 0.039).

Serum NGAL increased in the first several hours in response with CRRT treatment. However, there was a consistent trend of decrease in the serum NGAL throughout the treatment. The NGAL concentration in the ultrafiltration was between 16.9 and 22.7 ng/mL. NGAL levels were higher in non-survivors (197.4 ± 60.1 ng/mL) than those in survivors (107.1 ± 48.2 ng/mL) (P = 0.039) (Figure 3).

### Effect of CRRT on the mortality of severe AKI

The average number of dysfunctional organs in the non-survival group was much greater than in the survival group (chi-square statistics, χ^2^ > 6.63, P < 0.01). The mortality of patients increased as the number of dysfunctional organs increased. After CRRT treatment, the survival rate was 84.7% with 2 or 3 dysfunctional organs and 16.1% with ≥ 4 dysfunctional organs.

## DISCUSSION

The increased incidence of AKI is a known common postoperative complication after cardiac surgery [12]. In order to predict the morbidity and mortality of patients with severe AKI after cardiac surgery accurately, this study evaluated the effect of CRRT on the renal function, the hemodynamics, the pulmonary function, the cytokines and NGAL, combined with the APACHE II and MODS scoring system in the survival and the non-survival patients.

The CRRT is a process of slow, isotonic removal of water and solute, clearance of inflammatory mediators and myocardial depression factors, correction of electrolyte and acid-base imbalance, and maintenance of circulatory stability. Simultaneously, CRRT removes excessive fluid in the lung interstitium to improve lung function, increases blood pressure, and reduces carbon dioxide retention. In this study, there were 142 patients who required nutritional support and a large number of intravenous medications. We found that Cr and BUN in patients after CRRT treatment were much lower than those without CRRT treatment, indicating improvement of renal function. CRRT provides stable hemodynamics and was favorable for the recovery of renal function [13]. The renal function in 74.9% patients in the survival group became normal after CRRT. Our results confirmed previous report [6] that monocyte and endothelial cell function was improved and the ratio of anti-inflammatory to pro-inflammatory mediators was regulated, which reduces organ damage in patients with severe AKI who were treated with CRRT. The OI and MAP of patients treated with CRRT also improved from pretreatment levels.

Many studies have shown that levels of serum cytokines, such as TNF-α and IL-6, are increased in patients with severe AKI [14–16]. Pro-inflammatory cytokines may contribute to additional organ dysfunction, which is associated with high mortality [17]. TNF-α had a molecular mass approximately 17 kD, secreted by macrophages, mast cells, and natural killer cells, with the effect of stimulating the release of proinflammatory cytokines and prostaglandin inflammatory mediators from macrophages [18]. IL-6 is a 26-kD protein produced by the liver, lymphocytes, and activated macrophages. It is crucial in the acute-phase inflammatory response and is relevant to the inducers of cardiovascular disease [19]. CRRT may decrease inflammatory cytokine levels for patients with severe AKI [16]. In this study, the pro-inflammatory cytokines TNF-α and IL-6 were significantly reduced in the survival group compared with the non-survival group. A low level of pro-inflammatory cytokines is associated with low mortality. While the anti-inflammatory cytokineIL-10, did not change significantly between the two groups. It is suggested that IL-10 may appear before the rise of the anti-inflammatory cytokines, and CRRT might have blocked the progress of inflammation by decreasing inflammatory cytokines.

NGAL is expressed in a number of human tissues including gastrointestinal, respiratory, and urinary tissue and increases in response to inflammation [19]. It's been confirmed that NGAL is an early biomarker for kidney disease [20, 21]; therefore, NGAL may be considered an AKI biomarker in patients receiving CRRT. It had been demonstrated that NGAL provided protection against renal ischemic/reperfusion injury in animals [22]. In this study, NGAL is lower in patients in the survival group than that in non-survival group whose concentration in the ultrafiltration was 16.9-22.7 ng/mL.

However, the limitation of this study was that it was a one single-center analysis. A future multi-center study with a larger sample size and a longer follow-up period is required.

In summary, high levels of TNF-α, IL-6, and NGAL are associated with a poor prognosis in AKI. CRRT is a safe and effective treatment for patients with severe AKI after cardiac surgery. CRRT has been shown to improve the prognosis of patients who are treated in a timely manner. However, the optimal timing, dialysis protocol, and other aspects of the treatment of AKI with CRRT after cardiac surgery have not yet been determined and should be studied further.

## MATERIALS AND METHODS

### Patients and clinical characteristics

The study consisted of 153 patients (102 men and 51 women) who were in line with the diagnostic criteria for AKI following cardiac surgery treated with CBP between January 2005 and June 2014. Patients’ age ranges from 27 to 79 years (55.04 ± 13.27). Exclusion criteria included renal dysfunction before surgery, death within 24 hours after CBP and incomplete data. AKI is defined as serum Cr level that has doubled from baseline or urine output < 0.5 mL∙kg^-1^∙h^-1^ for > 12 h [23, 24]. MODS was defined according to the criteria adopted by the Academic Council for discussion and severity of disease staging score standards [11, 25]. Of the 153 patients, 91 patients had a New York Heart Association Functional Classification of III and 57 patients were class IV. Patients diagnosed with renal dysfunction before the surgery were excluded. Surgical procedures included 23 cases of mitral valve replacement, 7 cases of aortic valve replacement, 14 cases of double valve replacement (aortic valve and mitral valve), 37 cases of coronary artery bypass grafting, 19 cases of coronary artery bypass grafting and valve replacement, 11 cases of double valve replacement and tricuspid valvuloplasty, 7 cases of aortic dissection, 10 cases of cardiac tumor resection, and 23 cases involving complex, miscellaneous cardiac surgery. The average aortic clamp time was 83.97 ± 50.14 minutes. Postoperative vasopressors to maintain blood pressure were required in 129 patients, and 113 patients required mechanical ventilation. The Human Research Ethics Committee at Nanjing Drum Tower Hospital approved the study prior to its initiation. The patients and clinical characteristics of the survival and non-survival groups were summarized in Table 3.

**Table 3:** Comparison of clinical data of Group A (survival) and B (non-survival) (mean ± standard deviation)

	Sex (male/female, n)	Age (years)	CPB Time (min)	Time of CRRT away from the AKI (hours)	Time of CRRT away from urine <0.5 mL·kg^-1^·h^-1^ (hours)
Group A	61/28	55.03 ± 10.47	125.89 ± 38.47	12.81 ± 13.76	13.74 ± 6.19
Group B	43/21	53.39 ± 11.56	142.38 ± 50.29	33.98 ± 31.84	35.83 ± 32.71
*t*	0.401	0.265	-1.037	-2.316	-2.501
*P*	0.697	0.803	0.294	0.041	0.032

Group A (survival) had 89 cases and Group B (non-survival) had 64 cases. CPB, cardiopulmonary bypass; CRRT, continuous renal replacement therapy

### The CRRT method

Indwelling vascular access, with a double lumen catheter approach using a femoral or jugular vein and the Seldinger technique, was established in all 153 patients. The CRRT equipment used was the Baxter Aquarius/BM25 (Baxter, USA) and the AV600S hemofiltration device using a polysulfone membrane with an area of 1.4 m^2^ (Fresenius, St. Wendel, Germany). A Baxter continuous blood purification line was used for continuous venovenous hemofiltration. The average replacement fluid flow rate was 3000 mL/h with a blood flow of 160-200 mL/min using low molecular weight heparin as anticoagulant.

### Assays on serum cytokine

Serum samples were collected before CRRT (0h), and at 4, 8, 24, 48h after the beginning of CRRT and stored at -80°C for later cytokine assay. The concentrations of interleukin-6 (IL-6) and tumor necrosis factor-α (TNF-α) were measured by ELISA per the manufacturers’ directions. IL-6 and TNF-α anti-body were purchased from R&D Systems (Minneapolis, MN).

### Sampling and quantification of NGAL

Serum samples for quantification of NGAL were obtained from each patient before CRRT (0h), and at 4, 8, 24, 48 h after the beginning of CRRT. Samples were immediately centrifuged at 3000 g for 10 min, divided into aliquots, and stored at -80°C. NGAL was measured using a commercially available ELISA kit (BioPorto Diagnostics, Gentofte, Denmark) according to the manufacturer's instructions.

### Other clinical indices

All patients were given an APACHE II (acute physiology and chronic health evaluation II) and MODS score. The changes in heart rate (HR), mean arterial pressure (MAP), oxygenation index (PaO_2_/FiO_2_), white blood cell(WBC) count, platelet count (PLT), and renal function as indicated by the blood urea nitrogen (BUN) and creatinine (Cr) were all monitored and recorded during the hospital stay.

### Statistical analysis

Analyses were performed using SPSS v.13.0 (SPSS Inc., Chicago, IL, USA). Results are presented as mean ± standard deviation. Differences between groups were assessed using one-way ANOVA (followed by least-significant difference for multiple comparisons). Significance was set at P-value < 0.05 (two-tailed).
